# Transcatheter Aortic Valve-in-Valve Implantation in a Patient Due to the Degeneratation of the Biointegral Valve Conduit as a Result of Previous Infectious Endocarditis

**DOI:** 10.7759/cureus.69453

**Published:** 2024-09-15

**Authors:** Marco Aiello, Riccardo A Sansonetti, Giulia Magrini, Giovanna Pepe, Marco Ferlini, Elena Seminari

**Affiliations:** 1 Cardiac Surgery, Fondazione Istituto di Ricovero e Cura a Carattere Scientifico (IRCCS) Policlinco San Matteo, Pavia, ITA; 2 Cardiology, Fondazione Istituto di Ricovero e Cura a Carattere Scientifico (IRCCS) Policlinco San Matteo, Pavia, ITA; 3 Nuclear Medicine, Fondazione Istituto di Ricovero e Cura a Carattere Scientifico (IRCCS) Policlinco San Matteo, Pavia, ITA; 4 Infectious Diseses, Fondazione Istituto di Ricovero e Cura a Carattere Scientifico (IRCCS) Policlinco San Matteo, Pavia, ITA

**Keywords:** 18f-fdg pet/ct, enterococcus faecalis, prosthetic valve endocarditis, tavr, transcatheter aortic valve replacement, valve-in-valve, viv-tavr

## Abstract

Graft infection, fistula, and mediastinitis are reported among the serious cardiovascular complications after a Bentall procedure. Surgery associated with antimicrobial treatment is usually recommended but not easily feasible in most cases. In this report, we describe a case of successful valve-in-valve (ViV) transcatheter aortic valve replacement (TAVR) in a patient with a degenerated bioconduit from a previously healed infectious endocarditis (IE). The TAVR procedure has been demonstrated to be a therapeutic option in selected cases with a previous history of IE who have been fully treated with antimicrobial therapy and who present a low risk of local re-infection and are deemed at prohibitive or high risk for surgical replacement. Data on TAVR on a bioconduit after a Bentall procedure are scarce. The present case underlines that a long follow-up and individualized treatment could improve the prognosis in patients with a history of prosthetic valve and aortic graft infection and severe valve dysfunction who cannot undergo surgical treatment. The 18F-labeled fluoro-2-deoxyglucose positron emission tomography/computed tomography (18F-FDG PET/CT) result could be successfully employed in the decision algorithm. Long-term antibiotic treatment, which could be lifelong in some instances, could be a reasonable choice when the risk of recurrence is associated with the risk for the patient's life.

## Introduction

Current American College of Cardiology/American Heart Association guidelines [1] and 2023 European Society of Cardiology guidelines [2] recommend a combination of surgery and antibiotics as the gold standard treatment for infective endocarditis (IE) in patients with symptomatic heart failure, uncontrolled infection, or at high risk of septic emboli, but also in cases of severe residual valvular dysfunction. Transcatheter aortic valve replacement (TAVR) is the treatment of choice in patients with severe symptomatic aortic stenosis at prohibitive surgical risk as an alternative to surgical valve replacement (SAVR) in patients at high, intermediate, and low risk after a shared decision of the Heart Team. Performing TAVR in patients with active endocarditis can be at high risk of infection recurrence as the infected valve leaflets or abscesses are not removed. However, TAVR can be implanted in selected cases of patients with a previous history of IE, fully treated with antimicrobial therapy, and with a low risk of local infection [3, 4]. Furthermore, it should be considered that nearly one-quarter of patients with surgical indications do not undergo surgery because of high operative risk [5]. Graft infection, fistula, and mediastinitis are reported among the serious cardiovascular complications after a Bentall procedure. In these cases, surgery associated with antimicrobial treatment is usually recommended but not easily feasible in most cases [6].

In this report, we describe a case of successful valve-in-valve (ViV) TAVR in a patient who experienced a previously healed infectious endocarditis on a bioconduct, which got degenerated.

## Case presentation

**Figure 1:**
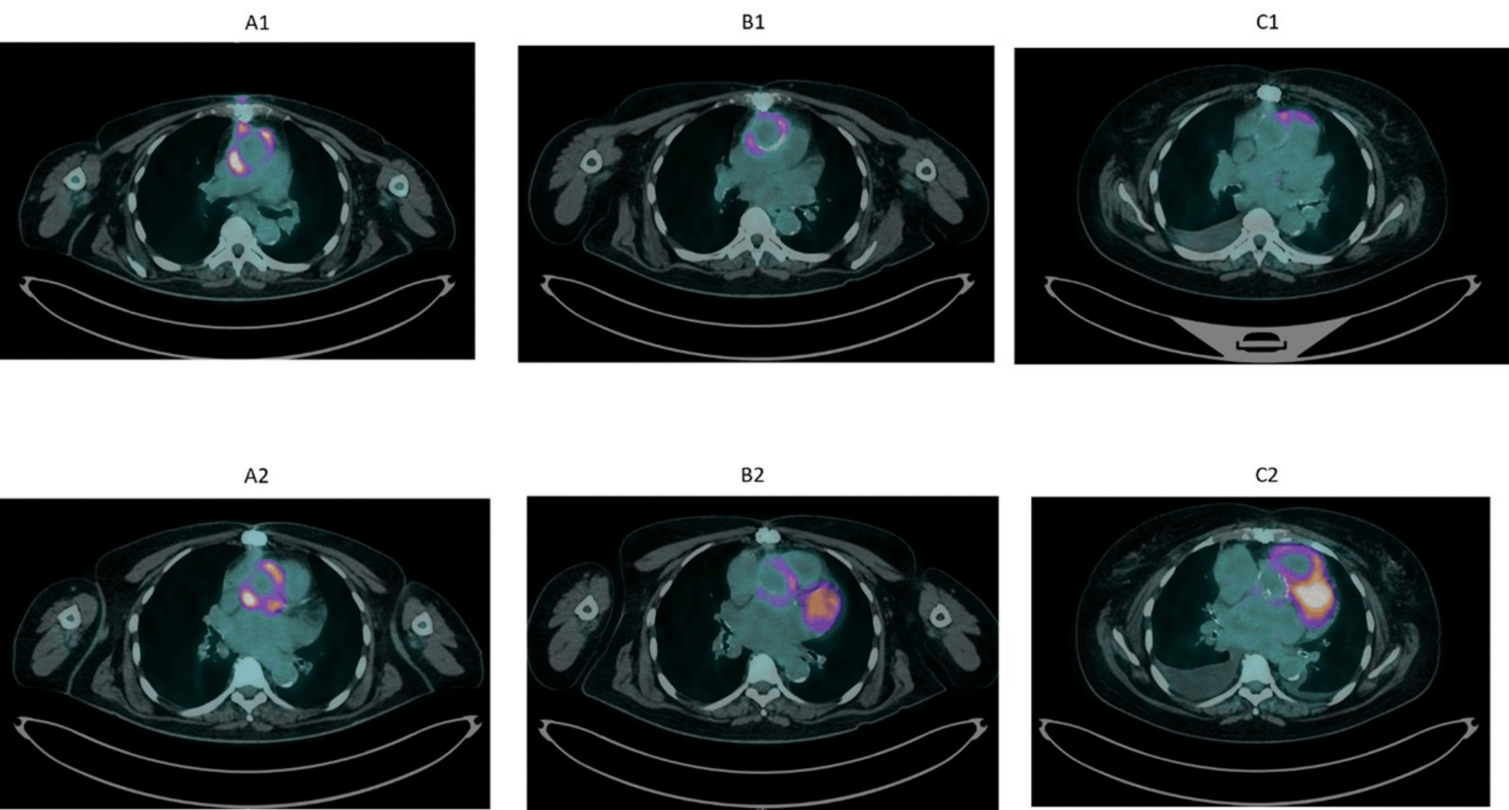
The remarkable tracer uptake around the vascular graft seen in 2021 (A1) decreases in 2022 (B1) up to normalization in 2023 (C1). Likewise the subcutaneous focus outside the sternum. Intense tracer uptake on the valve plan in 2021 (A2). Uptake reduction in 2022 (B2) and complete response to treatment in 2023 (C2). Bilateral pleural effusion in 2023 due to worsening of cardiac function, also highlited by the heart dilatation.

A 70-year-old female with a history of chronic kidney disease (Kidney Disease Improving Global Outcomes (KDIGO) stage IV), atrial fibrillation, arterial hypertension, and colon polyposis underwent a Bentall procedure in December 2019, using a 23 mm Biointegral valved conduit (BioIntegral Surgical, Inc. Mississauga, Ontario, Canada), for an ascending aortic aneurysm with concomitant severe bicuspid aortic valve stenosis. In February 2021, the patient was admitted to another hospital for fever and dyspnea and treated with vancomycin for an *Enterococcus faecalis* (*E. faecalis*) bacteremia episode. In May 2021, she was admitted to our hospital due to recurrent* E. faecalis* bacteremia. A transoesophageal echocardiogram (TOE) showed vegetation adhering to the bioprosthetic aortic valve cusps and pathologic tissue extending to the entire pericardial ascending aorta graft with an atrial septum thickening. Antimicrobial therapy was started, including ampicillin (dosage adjusted for renal function) plus ceftriaxone. Furthermore, a thoracic CT scan showed fluid collection in subcutaneous tissue above the sternal manubrium. A surgical approach was considered prohibitive considering the advanced age, the renal failure, the comorbidities, and the intrinsic risk linked to a re-do Bentall procedure (EUROSCORE II 30.47%). An 18F-labeled fluoro-2-deoxyglucose positron emission tomography/ computed tomography (18F-FDG PET/CT) (Figure 1A) confirmed the diagnosis of infection involving the aortic root, the ascending aorta, and the anterior mediastinum soft tissues.

The blood cultures resulted negative by day three of admission, and a subsequent TOE (on day 23) documented the disappearance of the valvular vegetation. After six weeks of parenteral antibiotic therapy, the patient was discharged on oral linezolid (600 mg twice a day (bid)) for two additional weeks and subsequently continued with oral amoxicillin according to renal function (1 g bid). She had persistently negative blood cultures on follow-up visits and continued long-term antimicrobial treatment. The TOEs were also persistently negative for IE lesions. After eight months, the follow-up 18F-FDG PET/CT scan showed a strong reduction of the pathologic tracer uptake (Figure 1B). Moreover, a focus of intense tracer uptake was described in the right side of the abdomen, corresponding to the colic wall, a suspected case of neoplastic disease, which was confirmed at subsequent investigations and identified as colon carcinoma. Therefore, the patient was surgically treated in July 2022 and underwent adjuvant chemotherapy until the end of 2022, with a clinical and radiologically complete response.

In August 2023, the patient presented with progressive heart failure symptoms. The TOE showed severe aortic valve regurgitation due to an intraprosthetic leak at the level of non-coronary cusp, associated with left-ventricular distension, with no signs of endocarditis. This resulted in the diagnosis of structural aortic bioprosthesis deterioration with healed IE. After a multidisciplinary discussion with the Heart Team, the surgical risk was confirmed as too high, and a ViV procedure was taken into account.

The 18F-FDG PET/CT repeated in November 2023 showed no relevant tracer uptake in the areas of the previous infection site (Figure 1C), as well as blood cultures and inflammation indices (white blood cell (WBC) count and C-reactive protein) were negative. Alongside 18F-FDG PET/CT negativity from the infective point of view, moderate heart dilatation was also evident. As determined by preoperative CT scan, the annulus dimensions were 23 × 24 mm, the sinus of Valsalva dimensions were 26 × 25 × 25 mm, the ascending aorta diameter was 41 mm distally to the aortic prosthesis, and the distance between the annulus and the left coronary artery ostium was 13 mm, whereas the distance with the right coronary artery ostium was 18 mm (imagine TAC1; imagine TAC2). No annular calcification was described.

A cause of concern was the risk of recurrent IE, but the absence of tracer uptake areas in the 18F-FDG PET/CT made us more comfortable about the procedure. Considering the radiological and microbiological data and the absence of any evidence of infection, in February 2024, the patient underwent a ViV-TAVR with implantation of a 26-mm balloon-expandable SAPIEN 3 Ultra valve (Edwards Lifesciences, Irvine, CA). The ViV-TAVR was performed using the right transfemoral approach under general anesthesia and with TOE guidance (Video 1). The procedure was completed without any early complications, such as stroke or vascular injury. Immediately after implantation, echocardiography confirmed the correct positioning of the valve without any significant transvalvular gradient or paravalvular leak (supplement material). She underwent periprocedural antibiotic prophylaxis with cefazolin and continued amoxicillin. Post-procedural complications included acute kidney injury in chronic kidney disease and atrial fibrillation recurrence. After medical optimization, the patient was discharged on day 18. Six months after the procedure, the patient's condition is fine, she performs her normal duties, and there is no evidence of endocarditis recurrence.

**Video 1:** Valve-in-valve transcatheter aortic valve replacement performed on the patient This video has been recorded by the authors.

## Discussion

The TAVR procedure has been demonstrated to be a therapeutic option in selected cases with a previous history of IE who have been fully treated with antimicrobial therapy and who present a low risk of local re-infection and are deemed at prohibitive or high risk for surgical replacement [3, 4, 7, 8]. While TAVR on bio-prosthetic aortic valves has been widely described, only one case of TAVR for treating bio-prosthetic Bentall conduit failure after IE has been reported [9]. The biointegral valved conduit is a stentless and all-biologically composed bioconduit, composed of a porcine valve and a single layer of bovine pericardium; its use is generally associated with a low infection rate [10]. However, if prosthetic bioconduit endocarditis occurs, we demonstrate the feasibility of the TAVR procedure in the setting of a healed IE in patients with a fully biological aortic valve-aortic conduit. An important challenge of this case was the absence of landmarks (no calcifications, stentless conduit, and fully biological valve) to guide the positioning of the new prosthesis. In addition, ViV-TAVR in stentless surgical prostheses is associated with an increased risk of dislocation and coronary obstruction. The risk of coronary obstruction in this case was not significant for the adequate distance between the annulus and the coronary ostia. Based on our experience with balloon-expandable prostheses, we used a SAPIEN 3 Ultra valve. The most frequent complication in cases of prosthetic failure after IE treated with TAVR remains infection. Recurrent infections are associated with an increased risk of death [4], and it is of straightforward importance to exclude active infection before the TAVR procedure to reduce the risk of complications [4].

Length of antimicrobial treatment of vascular graft infections that cannot undergo surgical treatment despite indication remains to be defined [11]. The treatment duration from the initial IE diagnosis to TAVR is heterogeneous, from six weeks to several months in the case of prosthetic valve IE [4] or also long life in the case of previous Bentall procedures [6, 11]. Therefore, the length of treatment per se cannot be the unique parameter to rely on in the decision process. The 18F-FDG PET/CT could be a useful tool to support the evidence of the absence of infection. A positive 18F-FDG PET/CT has been introduced among the major diagnostic criteria for infective prosthetic valve endocarditis [12]. While PET has a relatively limited role in the assessment of native valve endocarditis, according to the literature, its sensitivity and specificity in prosthetic valve endocarditis are 73%-100% and 71%-100%, respectively. The 18F-FDG PET/CT improves the sensitivity of the modified Duke criteria from 52%-70% to 91%-97%. [13].

In addition, 18F-FDG PET/CT appears to be a promising diagnostic tool to guide the timing of suspension of antibiotic therapy in patients who cannot be operated on and are treated with long-term antimicrobial treatment [6, 11], despite rare cases of infection recurrence after a negative 18F-FDG PET/CT in patients with aortic graft infections that have been described [11].

The 18F-FDG PET/CT shows high sensitivity (93%) in excluding possible infections in case of suspicion of infection of either aortic valve or tube graft [13], and could be a powerful instrument in identifying the patients at lower risk of infections in case of TAVR procedure in selected patients deemed at unacceptably high risk for traditional surgical procedure. In the present case, an 18F-FDG PET/CT resultant negative for infective foci before the TAVR procedure, associated with negative blood cultures and negative inflammation indices, was fit to exclude an active prosthetic and graft infection before TAVR procedures. As the patient remains at risk for *E. faecalis* bacteremia due to colon polyposis and previous cancer, we chose a lifelong treatment with amoxicillin.

## Conclusions

The present case underlines that a long follow-up and individualized treatment could improve the prognosis in patients with a history of prosthetic valve and aortic graft infection and severe valve dysfunction that cannot undergo surgical treatment. The result of 18F-FDG PET/CT could be successfully employed in the decision algorithm. Long-term antibiotic treatment, which could be lifelong in some instances, could be a reasonable choice when the risk of recurrence is associated with the risk for the patient's life.
